# Hereditary angioedema with normal C1 inhibitor in a French cohort: Clinical characteristics and response to treatment with icatibant

**DOI:** 10.1002/iid3.137

**Published:** 2017-01-11

**Authors:** Laurence Bouillet, Isabelle Boccon‐Gibod, David Launay, Anne Gompel, Gisele Kanny, Vincent Fabien, Oliver Fain, for the IOS Study Group

**Affiliations:** ^1^ Department of Internal MedicineNational Reference Centre for Angioedema (CREAK)Université Grenoble Alpes, Joint Unit 1036 INSERM‐CNRS‐CEACHU GrenobleFrance; ^2^ U995 Lille Inflammation Research International Center (LIRIC)University of LilleLilleFrance; ^3^ Inserm, U995LilleFrance; ^4^ département de médecine interne et immunologie cliniqueCHU LilleLilleFrance; ^5^ National Reference Centre for Angioedema (CREAK)LilleFrance; ^6^ Gynaecology and Endocrinology UnitParis Descartes University, Cochin‐Port Royal HospitalParisFrance; ^7^ EA 7299 Internal Medicine, Clinical Immunology and AllergologyLorraine University, University HospitalVandoeuvre‐lès‐NancyFrance; ^8^ ShireZugSwitzerland; ^9^ Department of Internal MedicineSaint‐Antoine Hospital (AP‐HP)Paris 6 University, DHUi2BParisFrance

**Keywords:** hereditary angioedema, icatibant, normal C1 INH

## Abstract

**Introduction:**

The clinical characteristics and icatibant‐treatment outcomes of patients with hereditary angioedema with normal C1 inhibitor (HAE‐nC1 INH) are limited.

**Methods:**

We retrospectively analyzed data from French HAE patients enrolled in the Icatibant Outcome Survey registry (from July 2009 to September 2013) to compare disease characteristics and the effectiveness and safety of acute icatibant‐treated angioedema attacks in patients with HAE‐nC1 INH, HAE with C1 INH deficiency (type I), or dysfunction (type II).

**Results:**

One center in Grenoble contributed 22 patients with HAE‐nC1 INH and a family history of HAE while 15 centers across France contributed 153 patients with HAE type I and seven patients with HAE type II. Patients with HAE‐nC1 INH compared to HAE type I, respectively, were more likely to be female (88.1% vs. 63.4%), older at median age of disease onset (21 years vs. 15 years), and have a greater rate of abdominal (80% vs. 61%) and laryngeal (23% vs. 14%) attacks. Icatibant was effective in both groups though the median time to resolution of attack was significantly longer in the HAE‐nC1 INH group (20.0 h, 37 attacks) versus the HAE type I group (14.0 h, 67 attacks). Icatibant was self‐administered for 96.1% of attacks in patients with HAE‐nC1 INH and 75.8% in patients with HAE type I. No serious adverse side effects related to icatibant were reported.

**Conclusions:**

These data help further define the disease characteristics of HAE‐nC1 INH in the French population and extend the limited data reporting the safe and effective use of icatibant in acute treatment of angioedema in French patients diagnosed with HAE‐nC1 INH.

## Introduction

Hereditary angioedema (HAE) is a rare disease affecting approximately one in 50,000 patients 1, 2 and is characterized by recurrent and unpredictable attacks of edema, predominantly localized to the skin, gastrointestinal tract, extremities, and upper airway. The two most common forms of HAE, caused by mutations of the *SERPING1* gene, are characterized by a deficiency (type I) or non‐functionality (type II) of C1 inhibitor (C1 INH) 3, 4. However, some patients have a distinct form of HAE that is not associated with C1 INH abnormalities, HAE with normal C1 INH (HAE‐nC1 INH) 5, 6. This condition shares many of the clinical manifestations of HAE with C1 INH deficiency, including potentially fatal laryngeal swelling attacks 7, 8. Recently, international consensus guidelines cataloguing the diagnosis and treatment of HAE‐nC1 INH have been published 4, 9.

A diagnosis of HAE‐nC1 INH can be made by an identification of two known mutations in the coagulation factor XII gene. Dewald et al. 10 identified factor XII mutations in HAE‐nC1 INH patients. The more common factor XII mutation causing HAE‐nC1 INH is 1032C → A (Thr309Lys) 10, 11. A rarer factor XII mutation causing HAE‐nC1 INH is 1032C → G (Thr309Arg) 9, 10. These mutations are useful as a diagnostic aid in approximately 20% of patients 8. Other patients with HAE‐nC1 INH have an unknown genetic cause, but can be identified through laboratory testing of plasma concentration or functionality of C1 INH, lack of effectiveness of long‐term prophylaxis with high doses of anti‐histamines, and a positive family history of angioedema 9, 12.

An additional diagnostic indicator is the involvement of estrogens as a potential attack trigger 7. The precise prevalence of HAE‐nC1 INH has to date been confounded by a reliance on clinical diagnosis and the lack of a validated diagnostic test 13, 14. However, several groups have reported disease characteristics in patients diagnosed as HAE‐nC1 INH from Germany 15, Spain 16, 17, Italy 18, and France 8.

In the absence of robust clinical trial data and treatments approved by regulatory authorities, treatment of HAE‐nC1 INH is based on clinical experience with the more common forms of HAE with C1 INH deficiency and with therapies that either directly or indirectly modulate bradykinin metabolism 4. Icatibant, a bradykinin‐2 receptor antagonist, is approved in the EU, the US, and some other countries and regions for symptomatic treatment of acute HAE attacks in patients with HAE with C1 INH deficiency based on data from three Phase III trials 19, 20. Although icatibant is not approved for use in patients with HAE‐nC1 INH, case reports and series describing the off‐label use of icatibant in these patients have previously been reported 17, 21, 22.

The Icatibant Outcome Survey (IOS) is an international, prospective observational study documenting safety and clinical outcomes for HAE patients who received at least one dose of icatibant (NCT01034969). The IOS thus provides an opportunity to evaluate icatibant treatment outcomes in patients with HAE‐nC1 INH in the real‐world setting. The aim of this study was to compare the disease characteristics and outcomes of acute icatibant‐treated angioedema attacks between patients with HAE‐nC1 INH in Grenoble center (France) and patients with HAE with C1 INH deficiency (type I) or dysfunction (type II) in the French population.

## Materials and Methods

The IOS is conducted in accordance with the Declaration of Helsinki and the International Conference on Harmonisation of Good Clinical Practice Guidelines. The protocol was approved by local ethics committees and/or health authorities, and patients provided written informed consent. Analyses included data from French patients enrolled in IOS with a diagnosis of HAE with C1 INH deficiency (type I) or dysfunction (type II) and HAE‐nC1 INH who had been treated with HCP‐ or self‐administered icatibant between July 2009 and September 2013.

**Figure 1:**
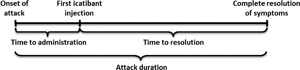
Definition of attack outcomes.

The design of IOS has been described previously 23, 24, 25, 26, 27, 28. In brief, symptomatic patients were diagnosed at enrollment with HAE type I (C1 INH deficiency) or II (normal levels of C1 INH, but with dysfunctional protein). The distinction between types I and II HAE was made on the basis of family history and HAE type II was diagnosed in symptomatic patients by testing C1 INH concentration and function. In this case, if C1 INH level was normal or above normal (≥15–50 mg/dL) and function was below normal levels (i.e., <70–130%), then patients received an HAE type II diagnosis 27. For HAE‐nC1 INH (normal C1 INH level and function), a diagnosis was made in symptomatic patients if C1 INH level was normal or above normal (≥15–50 mg/dL) and function was normal or above normal levels (i.e., ≥70–130%). In addition, for an HAE‐nC1 INH diagnosis, there was evidence that conventional treatment with antihistamines and corticosteroids was unsuccessful, and there was either a confirmed Factor XII mutation or family history of HAE 9. All data from patients was collected at the time of enrollment and follow‐up visits at 6‐month intervals 28. Outcome measures included patient demographics and angioedema attack characteristics, including frequency, location, and severity of attack. In the IOS, attack severity was either classified as mild (i.e., only results in a mild interference in day‐to‐day activities), moderate (i.e., causes moderate interruption with day‐to‐day activities without the need for other countermeasures), severe (i.e., the effect on daily activities is severe and may require other countermeasures), or very severe (i.e., the effect on daily activities is very severe, and countermeasures are necessary). Icatibant treatment outcomes included validated measures of time to administration (from symptom onset to first subcutaneous icatibant injection), time to resolution (duration from icatibant injection to complete symptom resolution), and attack duration (time from symptom onset to complete symptom resolution) (Fig. 1). Treatment outcome data are reported from patients who provided complete information on time to administration, time to resolution, and duration of attack. Safety and tolerability of icatibant treatment were evaluated as per MedDRA (Version 12).

### Statistical analysis

Patient demographics, attack characteristics, and safety outcomes are summarized descriptively. Comparison of time to administration, time to resolution, and attack duration was performed using a mixed‐model analysis of repeated measures (Proc Mixed: SAS Institute, Cary, NC, USA). The Chi‐square test was used for the comparison of percentages and the level of statistical significance selected was *α* = 0.05.

## Results

One hundred and fifty‐three patients and seven patients from 15 centers across France had HAE with C1 INH types I and II, respectively, while 22 patients from a single site in Grenoble (France) had HAE‐nC1 INH. The number of patients recorded with deficient C1 INH type I (84%) is greater than the number with type II (4%) or normal C1 INH (HAE‐nC1 INH) (12%). Previously reported proportions for type I, type II, and normal C1 INH are about 85%, 15%, and <1%, respectively 23, 25. Obtaining equal numbers of HAE patients is both impractical and an unrealistic representation of the HAE population. Interestingly, in this French cohort, the number of patients with HAE‐nC1 INH outweighed those with type II. The IOS registry, however, is ongoing, and so in the future, these numbers may vary as more accurate HAE diagnoses are made. Patients with a diagnosis of HAE‐nC1 INH were predominantly female (81.8%). Patient demographics are described in Table 1. All 22 symptomatic patients in this study who were identified to have normal C1 INH, had a confirmed family history of HAE but did not have a genetic C1 INH deficiency suggesting that they had normal C1 INH activity in their plasma. Sixteen of these patients were tested for the presence of a Factor XII (FXII) mutation and four of these patients were confirmed as having this mutation (confirmed as a large deletion or rearrangement in the FXII gene) and nine of these patients were found to not have the FXII mutation. Out of the remaining 3/16 patients that were tested for the FXII mutation, one patient had a single nucleotide polymorphism (c.2399C>A) in the gene encoding aminopeptidase P that has previously been shown to be associated with increased levels of bradykinin 29, one patient did not have a large deletion or rearrangement in the FXII gene but had the p.Thr328Lys variant in FXII which is associated with higher FXII amidolytic activity 11, 30, and one patient had an unknown genetic cause.

**Table 1:** Patient demographics

		HAE type I, *n* = 153 (%)	HAE type II, *n* = 7 (%)	HAE with normal C1 INH^a^, *n* = 22 (%)
Gender, n (%)				
Male		56 (36.6)	5 (71.4)	4 (18.2)
Female		97 (63.4)	2 (28.6)	18 (81.8)
Median age at enrollment, years (IQR)		*n* = 15238.0 (27.5–51.6)	*n* = 7, 53.4 (28.4–55.2)	*n* = 22, 35.1 (28.0–42.8)
Median age at first symptoms, years (IQR)		*n* = 115, 15.0 (8.0–20.0)	*n* = 5, 20.0 (15.0–23.0)	*n* = 19, 21.0 (16.0–29.0)
Median age at diagnosis, years (IQR)		*n* = 132, 20.3 (13.9–33.6)	*n* = 6, 31.8 (27.5–49.7)	*n* = 21, 29.4 (23.5–40.2)

IQR, Interquartile range.

^a^ Off‐label use.

### Attack characteristics

**Figure 2:**
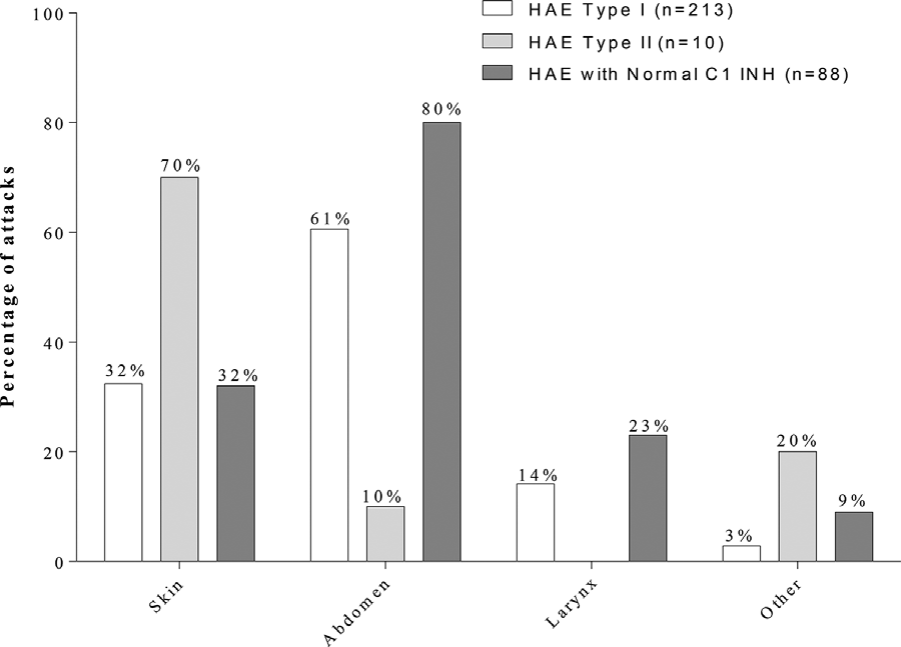
Frequency of attack location for attacks that are treated with icatibant. An attack can affect multiple locations. Missing affected sites are not shown.

Data enabling evaluation of icatibant‐treated attack characteristics was available for 61 patients (214 attacks) with HAE with C1 INH deficiency (type I), three patients (11 attacks) in the HAE with C1 INH dysfunction (type II) group, and 10 patients (90 attacks) in the HAE‐nC1 INH group (Table 2). The proportion of severe/very severe attacks was lower for patients with HAE type I (86.4%) than HAE‐nC1 INH (94.7%). Attack characteristics were only recorded for two out of 11 attacks in HAE type II patients, one of which was classed as severe or very severe. Icatibant was predominantly self‐administered in both the HAE‐nC1 INH and HAE type I groups. A single icatibant injection was administered in 78.6% of attacks in patients with HAE type I with 18.9% of attacks requiring a second injection, while a single icatibant injection was administered in 70.0% of attacks in patients with HAE‐nC1 INH with 24.0% of attacks requiring a second injection. Patients with HAE‐nC1 INH compared with patients with HAE type I reported a greater percentage of attacks localized to the abdomen (80.0% vs. 61.0%, respectively) and larynx (23.0% vs. 14.0%, respectively). Patients with HAE type II experienced attacks predominantly involving the skin (70.0%) and had no attacks on the larynx (Fig. 2).

**Table 2:** Attack characteristics for attacks treated by icatibant

Parameter		HAE type I, (*n* = 153)	HAE type II, (*n* = 7)	HAE with normal C1 INH^a^, ^b^,(*n* = 22)
Number of patients		61	3	10
Number of attacks		214	11	90
Severe/very severe attacks^d^, %		86.4	50.0	94.7
Icatibant administration, %				
HCP		24.2	0.0	3.9
Self‐administered		75.8	100	96.1
Number of icatibant injections, %				
One		78.6^c^	100	70.0
Two		18.9	0	24.4
Three		2.4	0	5.6

^a^ Off‐label use.

^b^ Patients with a confirmed family history of HAE.

^c^ One patient with HAE with C1 INH deficiency typically administered icatibant at the prodromal stage, commonly using further injections as the attack started. Excluding this patient, 83.4% of attacks in patients with HAE with C1 INH deficiency required only one icatibant injection.

^d^ Data missing for 45 HAE type I attacks and nine HAE type II attacks.

### Icatibant treatment outcomes

#### Time to administration

**Figure 3:**
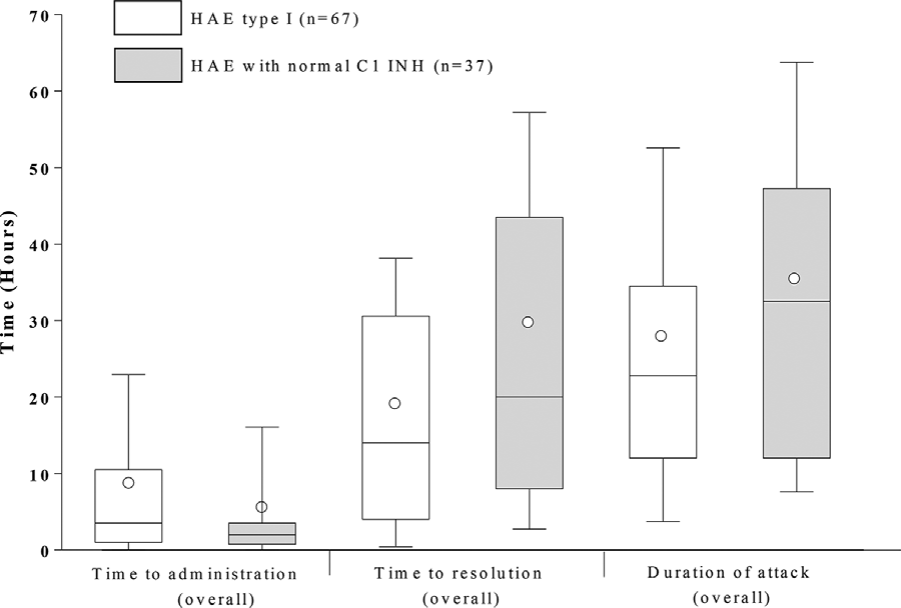
Treatment outcomes using icatibant. Time to administration, resolution, and attack duration (Overall). Box = IQR, whiskers = standard deviation, horizontal line = median, open circle = mean; Time to events were calculated only for attacks with complete data for time to administration, time to resolution, and attack duration. Note that no data on attack duration, time to administration, and time to resolution for HAE type II patients were recorded in the database.

Data for time to administration, time to resolution, and attack duration were available for 28 patients with HAE type I (67 attacks) and five HAE‐nC1 INH patients (37 attacks). Patients with HAE‐nC1 INH reported a faster median time to administration with a median (Q1, Q3) of 2.0 h (0.8, 3.5) compared with 3.5 h (1.0, 10.5) for patients with HAE type I, respectively, however, this difference was not significant (*P* = 0.233; Fig. 3). Time to resolution was significantly faster for patients with HAE type I compared to the HAE‐nC1 INH patients with a median (Q1, Q3) time to resolution of 14.0 h (4.0, 30.6) h versus 20 h (8.0, 43.5) h, respectively (*P* = 0.021; Fig. 3). Duration of attack was similar between both patients with HAE type I compared to the HAE‐nC1 INH patients with a median (Q1, Q3) attack duration of 22.75 h (12.0, 34.5) h versus 32.5 (12.0, 47.3) h, respectively (*P* = 0.157; Fig. 3).

#### Time to resolution by attack location

**Figure 4:**
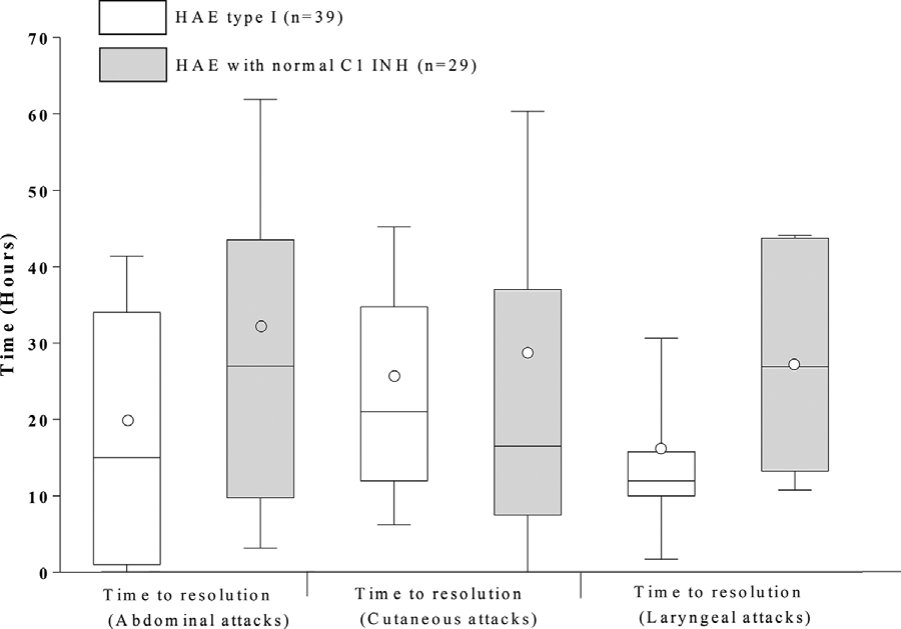
Time to attack resolution following administration with icatibant by attack location for Abdominal, Cutaneous, and Laryngeal attacks. Box = IQR, whiskers = standard deviation, horizontal line = median, open circle = mean. Time to events were calculated only for attacks with complete data for time to administration, time to resolution, and attack duration. Note that no data on attack duration, time to administration, and time to resolution for HAE type II patients were recorded in the database.

Time to resolution of attacks localized to the abdomen was available for 16 patients with HAE type I (39 attacks) and five patients (29 attacks) with HAE‐nC1 INH. The median (Q1, Q3) time to resolution in patients with HAE type I versus HAE‐nC1 INH was 15.0 (1.0, 34.0) h versus 27.0 (9.75, 43.5) h, respectively (*P* = 0.048; Fig. 4). Time to resolution of cutaneous attacks was available for 16 patients with HAE type I (29 attacks) and two patients (14 attacks) with HAE‐nC1 INH. The median (Q1, Q3) time to resolution in patients with HAE type I versus HAE‐nC1 INH was 21.0 (12.0, 34.8) h versus 16.5 (7.5, 37.0) h, respectively (*P* = 0.697; Fig. 4). Resolution of laryngeal attacks was available for nine patients with HAE type I (nine attacks) and three patients (12 attacks) with HAE‐nC1 INH. The median (Q1, Q3) time to resolution in patients with HAE type I versus HAE‐nC1 INH was 12.0 (10.0, 15.8) h versus 26.9 (13.3, 44.1) h, respectively (*P* = 0.125; Fig. 4).

#### Time to resolution by attack severity

**Figure 5:**
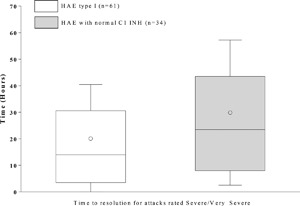
Time to resolution following icatibant administration for attacks rated “Severe/Very Severe.” Box = IQR, whiskers = standard deviation, horizontal line = median, open circle = mean. Time to events were calculated only for attacks with complete data for time to administration, time to resolution, and attack duration. Note that no data on attack duration, time to administration, and time to resolution for HAE type II patients were recorded in the database.

Time to resolution of attacks deemed to have either a severe or very severe impact on daily function was available for 24 patients with HAE type I (61 attacks) and five HAE‐nC1 INH patients (34 attacks). The median (Q1, Q3) time to resolution in patients with HAE type I versus HAE‐nC1 INH was 14.0 (3.5, 30.6) h versus 23.5 (8.0, 43.5) h, respectively (*P* = 0.051; Fig. 5).

#### Safety and tolerability

A total of 23 patients with HAE type I reported 35 adverse events (AEs) (Table 3). Of these, five AEs from two patients were considered related to icatibant treatment. For patients with HAE type II, a total of seven patients reported only one adverse event in one patient, none of which were considered related to the study drug. In the HAE‐nC1 INH group, 11 patients reported 44 AEs. Of these, three AE from two patients were considered related to icatibant treatment. No serious AEs related to icatibant treatment were reported for either group and no deaths were reported.

**Table 3:** Summary of adverse events

	HAE type I (*n* = 153 patients)		HAE type II (*n* = 7 patients)		HAE with normal C1 INH^a^ (*n* = 22 patients)	
	Patients	Events	Patients	Events	Patients	Events
Number of AEs	23	35	1	1	11	44
Number of AEs related to icatibant^b^	2	5	0	0	2	3
Drug ineffective	1	1	0	0	2	3
Blood pressure decreased	1	4	0	0	0	0

AE, adverse advent.

^a^ Off‐label use.

^b^ A missing relationship to icatibant has been considered as related to icatibant.

## Discussion

We compared demographic, clinical characteristics, and icatibant‐treatment outcomes of French patients with HAE with C1 INH deficiency or dysfunction (types I and II) and those with HAE‐nC1 INH.

The results confirm previous findings that HAE‐nC1 INH predominantly affects women 6, 14. The earlier age of onset of symptoms in patients with HAE with C1 INH deficiency correspond with data reported from 193 French patients 31. The later onset of clinical symptoms in patients in the HAE‐nC1 INH group, are similar to those reported in a series of 138 patients in Germany 15.

Patients with HAE‐nC1 INH differed significantly from patients with HAE with C1 INH deficiency or dysfunction (types I and II) with respect to an increased rate of abdominal attacks in the former group. This is a higher proportion than that reported by Bork et al. 15. A higher number of laryngeal attacks were noted in the HAE‐nC1‐INH group and confirms previous data 21. It is important to note that the IOS also registered angioedema attacks in other patients that were associated with conditions such as acquired angioedema (i.e., unassociated with hereditary angioedema), but these are not within the remit of this study. Figure 2 represents data on attacks that are treated with icatibant and does not include information on untreated attacks as these only involved a limited number of patients and is not within this study's remit.

The icatibant treatment outcomes in this study are consistent with previous reports of small case series in patients with HAE‐nC1 INH 17, 21, 22. The present study confirms and extends our previously reported open‐label, prospective evaluation of the effectiveness of icatibant in the treatment of eight patients with HAE‐nC1 INH, and seven patients with HAE type I 21. In 2009, we first reported the resolution of symptoms within 1–2 h following acute icatibant treatment of abdominal attacks in three female HAE‐nC1 INH patients 22.

While HAE‐nC1 INH patients initiated icatibant treatment slightly earlier compared to patients with HAE type I, the former reported a significantly longer time to complete resolution of attack. At this time, we have no explanation for this difference. Severe attacks are more frequent in HAE‐nC1 INH patients. When we compared the duration of severe attacks between the two groups, we still found a longer time to complete resolution. The difference is almost significant (0.051). The IOS study is ongoing and new prospective data may help us to better understand this difference.

Safety outcomes of the use of icatibant in HAE‐nC1 INH patients are consistent with the established safety profile of icatibant in patients with HAE with C1 INH deficiency 19, 20 and previous reports of safety in patients with HAE‐nC1 INH 17, 21, 22.

Limitations of the IOS include the lack of random assignment to treatment and a comparator and the reliance on patient reporting in which mild or moderate attacks may be under‐reported. A further IOS limitation was the data collection process where individual patient data were collected for variable durations that were deemed to be appropriate by both the physician and the patient. While patients participated in the registry, data were collected during routine patient visits. Physicians were asked to enter comprehensive baseline data at the time of patient enrollment, to perform follow‐up assessments and to update patient data in the registry on an ongoing basis and at a minimum of 6‐month intervals. Other study limitations include the fact that HAE‐nC1 INH is diagnosed only clinically with no known biomarkers to date, and that four of these patients had a factor XII mutation, with the remaining of these patients identified as having a family history only.

Overall, this study provides real world data to further characterize HAE‐nC1 INH in the French population. These data support the hypothesis that although icatibant is not yet an approved drug for HAE‐nC1 INH, it may be a potentially safe and effective option for treating patients with this condition.

## Authors’ Contributions

Laurence Bouillet, Isabelle Boccon‐Gibod, David Launay, Anne Gompel, Gisele Kanny, and Oliver Fain conceived, developed, and approved this manuscript. Vincent Fabien performed the statistical analysis, developed, and approved the manuscript. Under the direction of the authors, David Lickorish PhD, CMPP, and Sally Hassan PhD of Excel Scientific Solutions provided writing assistance for this publication.

## Conflict of Interest

Professor Bouillet and Dr. Boccon‐Gibod have received honoraria and travel grants from CSL Behring, Pharming, Shire, and Novartis and their institute has received research funding from CSL Behring, Shire, and Novartis. Dr. Launay has received honoraria from CSL Behring, Shire, and ViroPharma and research funding from CSL Behring and Shire. Dr. Gompel has received honoraria from CSL Behring, ViroPharma, and Shire. Dr. Kanny has received honoraria from Shire and ViroPharma. Dr. Vincent Fabien was a full‐time employee of Shire, Zug, Switzerland. Dr. Olivier Fain has provided scientific consultation for CSL Behring, Shire, and ViroPharma.
